# Understanding university students’ AI ethical decision-making in academic contexts: a SOR - social cognitive perspective

**DOI:** 10.3389/fpsyg.2026.1817904

**Published:** 2026-07-23

**Authors:** Hao Deng, Minli Yang, Liling Huang, Zhixin Yang, Nan Jiang

**Affiliations:** 1 Faculty of Education, Guangxi Normal University, Guilin, Guangxi, China; 2 School of Management, Guangxi Minzu University, Nanning, Guangxi, China; 3 Fine Arts College, Guangxi Arts University, Nanning, Guangxi, China

**Keywords:** academic integrity, AI ethical decision-making, cognitive complexity, moral cognition, PLS-SEM

## Abstract

**Introduction:**

The integration of generative artificial intelligence into higher education has reshaped students’ academic practices and blurred normative boundaries around academic integrity. While prior research has focused mainly on attitudes and usage intentions, limited attention has been paid to students’ AI ethical decision-making in academic scenarios and its psychological mechanisms. This study examined the associations among AI ethics guidance, academic ethics course experience, moral cognition, cognitive complexity, and AI ethical decision-making. It tested whether moral cognition mediated the relationships between normative inputs and AI ethical decision-making and whether cognitive complexity strengthened these relationships. The study integrated the Stimulus–Organism–Response framework with Social Cognitive Theory and combined net-effect, configurational, and importance–performance analyses.

**Methods:**

Survey data were collected from 1,106 undergraduates at Chinese universities. Partial least squares structural equation modeling was used to test direct, mediating, and moderating relationships, complemented by fuzzy-set qualitative comparative analysis to identify configurational pathways to high AI ethical decision-making. Importance–performance matrix analysis identified intervention priorities.

**Results:**

AI ethics guidance (*β* = 0.334, *p* < 0.001) and academic ethics course experience (*β* = 0.336, *p* < 0.001) were positively associated with AI ethical decision-making. Both normative inputs were positively associated with moral cognition (*β* = 0.479 and 0.280, respectively; *p* < 0.001), which was positively associated with AI ethical decision-making (*β* = 0.215, *p* < 0.001). Significant indirect relationships were identified through moral cognition (indirect *β* = 0.103 and 0.060; *p* < 0.001). Cognitive complexity strengthened the relationships between the two normative inputs and AI ethical decision-making (*β* = 0.077 and 0.073; *p* < 0.05). The model explained 66.3% of the variance in AI ethical decision-making (R^2^ = 0.663). fsQCA revealed no single necessary condition; however, AI ethics guidance and academic ethics course experience consistently emerged as core conditions across high-performing configurations (overall consistency = 0.934; coverage = 0.793).

**Discussion:**

The findings advance academic integrity research by shifting attention from attitudes to scenario-based decision quality and clarifying the internalization mechanism through moral cognition. Practically, they highlight the complementary roles of technological prompts and academic ethics course experience in fostering students’ AI ethical decision-making.

## 1 Introduction

The rapid diffusion of artificial intelligence (AI) is reshaping university students’ learning processes and modes of academic production (Feuerriegel et al., 2024). Across tasks such as writing, paraphrasing, code generation, information retrieval, and synthesis, AI has evolved from a supplementary tool into an embedded component of routine learning environments (Fui-Hoon Nah et al., 2023; Kenthapadi et al., 2023). While this transformation enhances efficiency and resource accessibility, it also restructures the risk landscape of academic integrity in higher education. Controversies increasingly arise in gray areas where AI use is technically feasible yet normatively ambiguous, shifting concerns away from traditional forms of plagiarism or ghostwriting (Dhruv et al., 2024). Emerging scholarship suggests that AI is prompting a transition in academic integrity governance, from detecting misconduct to clarifying normative boundaries, reconstructing standards, and strengthening students’ judgment capabilities (Eke, 2023; Mortlock and Lucas, 2024; Yusuf et al., 2024).

In this context, the central issue of academic integrity is no longer whether students endorse integrity norms, but whether they can make defensible ethical decisions in concrete AI use scenarios (Ward et al., 2024). Questions concerning citation, verification, and disclosure in AI-assisted academic work require students to engage in real-time ethical trade-offs under conditions of ambiguity (Dhruv et al., 2024). Compared with traditional misconduct, AI-related controversies are more closely associated with contextual use, transparency, responsibility allocation, and evidentiary credibility (Vasylyshyna et al., 2024). Accordingly, the quality of students’ decision-making in complex situations becomes a more appropriate focal outcome. Although prior research has examined AI and academic integrity from perspectives such as governance responses, institutional policies, and student and faculty attitudes (Artyukhov et al., 2025; DiPaulo, 2022; Kumar et al., 2024), explanations of how students form high-quality ethical decisions in specific AI-related contexts remain limited.

At the student level, empirical studies have predominantly relied on attitudes, perceived risks, usage intentions, behavioral tendencies, or self-reported compliance judgments to characterize students’ engagement with AI (Gallent Torres et al., 2023; Gruenhagen et al., 2024). While these constructs help explain willingness to use AI or concern about misconduct risks, they are insufficient to capture variations in decision quality under ambiguous task conditions. Therefore, compared with general attitudes or behavioral intentions, AI ethical decision-making more directly reflects situational judgment quality and serves as a more suitable core variable for explaining academic integrity differences in the AI era (Biagini, 2025; Chang and Wong, 2025). In this study, AI ethical decision-making refers to students’ ability and performance in identifying normative boundaries, weighing responsibilities and consequences, and making defensible choices in AI-related academic integrity scenarios. This construct differs from moral cognition. Moral cognition refers to students’ understanding, endorsement, and internalization of academic integrity principles and AI-use-related ethical norms, whereas AI ethical decision-making refers to the observable quality of students’ choices and justifications in specific scenarios. Accordingly, moral cognition is conceptualized as the internal psychological mechanism, and AI ethical decision-making is conceptualized as the decision-performance outcome. Given the task-dependent nature of AI use and the susceptibility of self-report scales to social desirability bias, this study adopts scenario-based tasks and performance-based scoring to assess decision quality in realistic or quasi-realistic contexts.

Regarding underlying mechanisms, prior research has discussed normative sources in a fragmented manner. One stream emphasizes technological environmental cues, such as platform reminders and detection mechanisms (Clark, 2025; Klella and Mrghem, 2024), whereas another focuses on institutionalized educational inputs, including academic ethics course experience and formal policy communication (Biagini, 2025; Chang and Wong, 2025). In practice, students’ AI ethical decision-making is influenced by both types of normative inputs. However, few studies have integrated them within a unified explanatory framework. To avoid conceptualizing AI ethics guidance as a purely objective safeguard, this study centers on students’ perceived AI ethics guidance and their perceived experiences of academic ethics course experience, conceptualizing them jointly as dual-source normative inputs at the perceptual level.

To explain how these normative inputs translate into AI ethical decision-making, this study integrates the Stimulus–Organism–Response framework with Social Cognitive Theory to construct a process model linking normative input, internalization, and decision performance (Bandura, 1991; Russell and Mehrabian, 1974). In this model, perceived AI ethics guidance and academic ethics course experience constitute the stimulus layer, moral cognition represents the organism layer, and AI ethical decision-making serves as the response layer. These constructs are positioned at different theoretical levels. Perceived AI ethics guidance and academic ethics course experience represent external normative inputs; moral cognition represents students’ internalization of academic integrity principles and AI-use-related ethical norms; and AI ethical decision-making represents the scenario-based outcome reflected in students’ choices and justifications. The Stimulus–Organism–Response framework provides the structural logic connecting external cues, internal states, and behavioral outcomes, while Social Cognitive Theory explains how normative information is internalized through observation, cognitive processing, reflection, and self-regulation. Furthermore, given that AI-related academic integrity judgments involve multiple cues, blurred boundaries, and consequence trade-offs (Balalle and Pannilage, 2025), this study introduces cognitive complexity as a key boundary condition. Cognitive complexity captures individual differences in information integration and hierarchical judgment (O'keefe and Sypher, 1981), thereby shaping the strength of normative input effects.

Methodologically, this study adopts a multi-method integration strategy that combines net effect estimation, configurational analysis, and action prioritization. First, partial least squares structural equation modeling is employed to examine the direct relationships among perceived AI ethics guidance, academic ethics course experience, moral cognition, cognitive complexity, and AI ethical decision-making, as well as the mediating role of moral cognition and the moderating role of cognitive complexity (Sarstedt et al., 2021). Second, fuzzy-set qualitative comparative analysis is applied to identify multiple configurational pathways leading to high levels of AI ethical decision-making (Elliott, 2013). Third, importance–performance matrix analysis is conducted to determine practical intervention priorities (Streukens et al., 2017), thereby providing actionable evidence for curriculum optimization and the design of AI-based ethical guidance.

Specifically, this study aims to analyze the direct and indirect relationships among perceived AI ethics guidance, academic ethics course experience, moral cognition, cognitive complexity, and AI ethical decision-making, and to identify both configurational pathways and practical intervention priorities for improving students’ AI ethical decision-making.

Accordingly, this study addresses four research questions. First, do perceived AI ethics guidance and academic ethics course experience influence students’ AI ethical decision-making, and if so, how? Second, is this influence mediated through the internalization process reflected in moral cognition? Third, does cognitive complexity shape the strength of the relationships among normative inputs, moral cognition, and AI ethical decision-making? Fourth, are there multiple equivalent configurational pathways leading to high AI ethical decision-making, and what practical intervention priorities can be derived from these pathways?

This study makes three primary contributions. Theoretically, it integrates perceived AI ethics guidance and academic ethics course experience within a unified Stimulus–Organism–Response and Social Cognitive framework, constructing and testing a mechanism chain linking normative input, moral cognition internalization, and AI ethical decision-making. Methodologically, it operationalizes AI ethical decision-making through scenario-based performance measures and combines partial least squares structural equation modeling, fuzzy-set qualitative comparative analysis, and importance–performance matrix analysis to establish complementary evidence. Practically, it provides targeted implications for case-based training and stepwise guidance in academic ethics course experience, as well as for optimizing ethical prompts and citation verification reminders embedded in AI tools.

## 2 Literature review

### 2.1 AI ethical decision-making

AI ethical decision-making refers to individuals’ ability and performance in identifying normative boundaries, weighing responsibilities and consequences, and generating defensible judgments and justifications within AI-related academic contexts. It is conceptually distinct from moral cognition. Moral cognition concerns individuals’ understanding, endorsement, and internalization of ethical norms, whereas AI ethical decision-making concerns the quality of situated choices and justifications made in specific AI-related academic scenarios. Unlike moral attitudes or behavioral intentions, AI ethical decision-making emphasizes the quality of situational judgment, focusing on how individuals interpret norms and translate them into context-specific choices.

Within the domains of academic integrity and AI use, AI ethical decision-making becomes particularly salient (Jensen, 2020; Nucci and Gingo, 2010). AI-assisted learning simultaneously involves efficiency enhancement, rule compliance, and responsibility attribution. The introduction of generative AI further blurs boundaries surrounding paraphrasing, citation, source verification, and authorship, rendering compliance no longer a mechanical application of rules but a process requiring principled trade-offs and reasoned justification (Bittle and El-Gayar, 2025). Accordingly, AI ethical decision-making provides a more direct analytical lens for understanding differences in academic integrity performance among students in AI-mediated contexts.

Existing research on AI and academic integrity has largely examined academic misconduct, ethical attitudes, usage intentions, and normative awareness, with primary emphasis on whether students comply with rules or endorse relevant norms (Wiredu et al., 2024; Yusuf et al., 2024). While such studies are valuable for identifying risk factors and attitudinal tendencies, they seldom directly investigate how students interpret norms, allocate responsibility, and engage in ethical trade-offs within concrete academic scenarios. Moreover, prior research often discusses technological normative cues and institutional training separately, lacking a unified framework to explain how distinct normative sources influence decision performance through shared psychological mechanisms. In comparison, empirical inquiry into how students construct defensible judgments in specific academic tasks remains limited (Okaiyeto et al., 2023; Wood and Moss, 2024). In the context of AI deeply embedded in learning processes, explaining academic integrity behavior solely through attitudes or intentions is insufficient to capture the cognitive quality of actual decision-making (Arar et al., 2025). Therefore, positioning AI ethical decision-making as a central outcome variable is necessary to advance understanding of how external normative inputs shape students’ decision performance through internal psychological processes.

### 2.2 Theoretical framework

Normative judgment in specific AI-related academic tasks fundamentally results from the continuous interaction between external normative cues and individual cognitive processing. Based on this premise, the present study adopts the Stimulus–Organism–Response framework to integrate multi-source normative inputs, internal psychological mechanisms, and AI ethical decision-making within a unified explanatory pathway (Jacoby, 2002). In this framework, external normative cues function as stimuli that influence individuals’ internal states, which subsequently shape their AI ethical decision-making performance in situational tasks.

**Figure 1:**
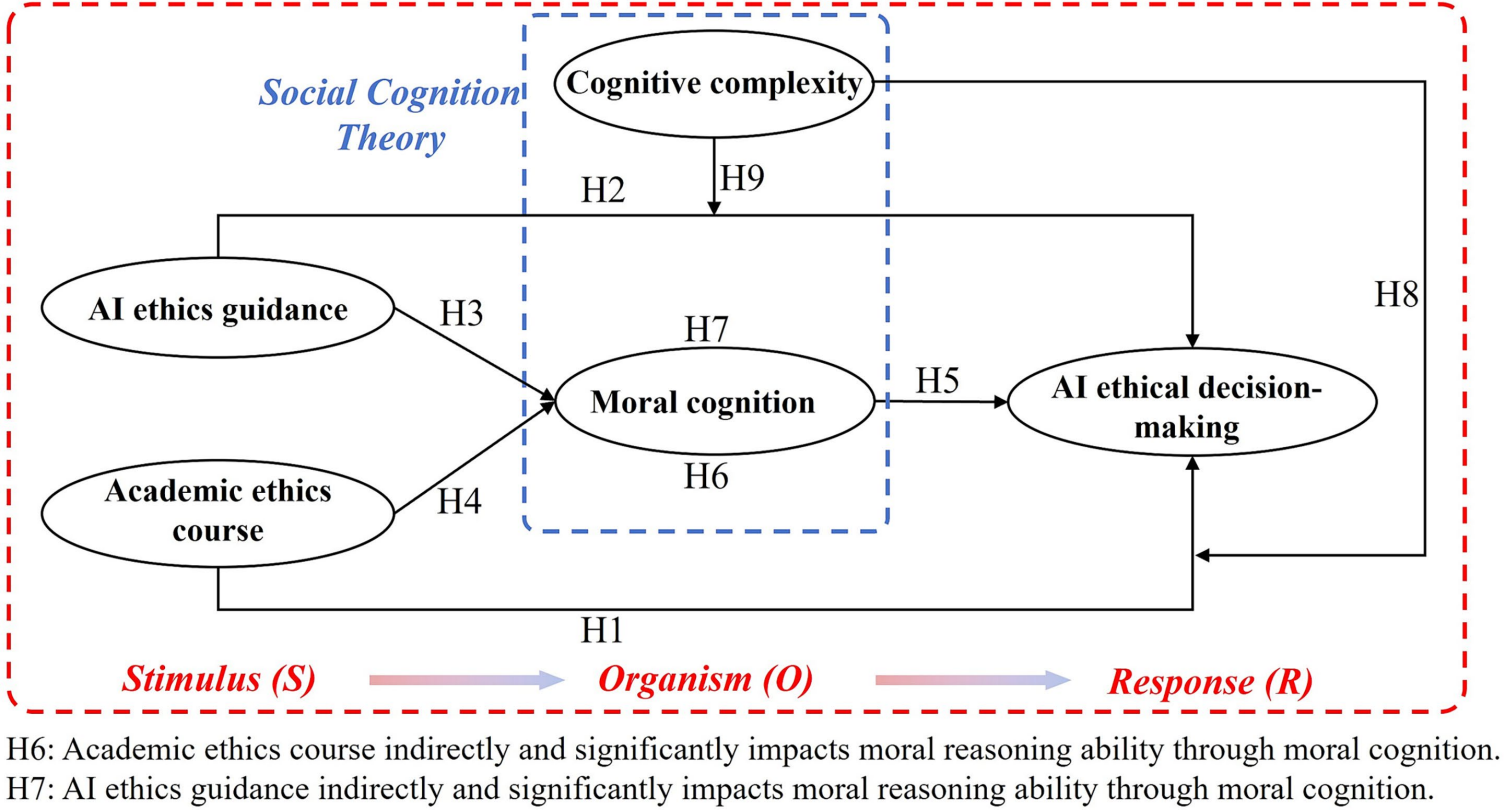
Theoretical framework.

To clarify how stimuli are transformed into internal states, this study incorporates Social Cognitive Theory as a complementary theoretical foundation. Social Cognitive Theory emphasizes the reciprocal interactions among environmental cues, cognitive processes, and behavior, and highlights the role of self-regulation in the internalization of norms (Schunk, 2012). From this perspective, the organism layer is conceptualized as moral cognition, defined as individuals’ understanding, endorsement, and internalization of academic integrity principles and AI compliance norms into internalized standards of conduct. Accordingly, external normative cues do not directly determine decision outcomes; rather, they exert influence through moral cognition and self-regulatory processes, thereby shaping students’ judgments and choices in specific contexts. The overall theoretical framework is illustrated in Figure 1.

At the stimulus level, this study further differentiates external normative sources into two types of normative socialization agents: technological agents and institutional agents. Technological agents are represented by AI ethics guidance embedded within task environments, providing real-time normative prompts and decision support (Braun et al., 2021). Institutional agents are reflected in academic ethics course experience, which promote normative understanding and internalization through systematic instruction, case-based training, and feedback mechanisms (Koplin, 2023). Together, these two agents constitute dual-source normative inputs influencing AI ethical decision-making, although their modes of influence may differ and potentially complement each other.

At the same time, individuals vary in their capacity to process normative cues. Cognitive complexity affects one’s ability to differentiate, integrate, and reflect upon multiple sources of information, thereby shaping the strength of the effects of dual-source normative inputs on moral cognition and AI ethical decision-making (Scott, 1962). Consequently, cognitive complexity provides an important theoretical basis for examining the boundary conditions under which normative inputs exert their influence.

### 2.3 Hypothesis development

#### 2.3.1 Academic ethics course experience

An academic ethics course experience refers to institutionally structured training through which universities systematically convey principles of academic integrity, normative requirements, and their application in concrete academic contexts. Prior research indicates that such courses, through case analysis, rule clarification, and feedback mechanisms, enhance students’ ability to identify academic misconduct and improve the quality of ethical judgment in complex academic situations (Benson and Enstroem, 2023).

Recent empirical studies further suggest that systematic ethics education not only reduces the incidence of misconduct but also strengthens students’ capacity for principled reasoning and justification in situations involving normative conflict, thereby enhancing AI ethical decision-making (Cahill and Mccabe, 2024; Huang et al., 2025). In the context of AI deeply embedded in learning processes, academic ethics course experience remain a critical institutional factor in shaping students’ AI ethical decision-making capabilities.

Accordingly, the following hypothesis is proposed:

*H1*: Academic ethics course experience has a significant positive effect on AI ethical decision-making.

#### 2.3.2 AI ethics guidance

AI ethics guidance refers to the integration of normative prompts, risk alerts, or ethical decision-support features into AI tools through technological interfaces within learning or writing contexts, thereby embedding academic norms directly into the AI usage process (Holmes and Miao, 2023; Kasneci et al., 2023). With the widespread adoption of generative AI in higher education, research has shown that students frequently encounter blurred ethical boundaries in AI-assisted writing and learning. Reliance solely on rule awareness is often insufficient to sustain high-quality judgment, whereas contextualized prompts can significantly enhance sensitivity to ethical considerations and improve decision quality (Lund et al., 2025).

Further empirical evidence indicates that when AI systems provide explanatory feedback and responsibility cues, learners demonstrate stronger normative understanding and more refined responsibility judgments in complex tasks, suggesting that technology-based ethical guidance contributes to more mature AI ethical decision-making (Cotton et al., 2024; Kasneci et al., 2023). In the context of generative AI deeply embedded in academic activities, AI ethics guidance may therefore strengthen situational normative cues and promote the development of students’ AI ethical decision-making.

Accordingly, the following hypothesis is proposed:

*H2*: AI ethics guidance has a significant positive effect on AI ethical decision-making.

#### 2.3.3 The mediating role of moral cognition

Moral cognition refers to individuals’ understanding, endorsement, and internalization of academic integrity norms and their ethical implications. It is conceptually different from AI ethical decision-making. Moral cognition represents the internal psychological mechanism through which external normative cues are transformed into relatively stable evaluative standards, whereas AI ethical decision-making represents the quality of students’ choices and justifications in specific academic scenarios. Prior research indicates that in learning environments where generative AI is deeply embedded, students’ ability to accurately identify ethical risks and comprehend the underlying logic of responsibility significantly affects subsequent decision quality (Mannuru et al., 2025).

From a technological perspective, AI ethics guidance enhances the visibility and processability of ethical information through normative prompts and risk alerts, thereby facilitating learners’ comprehension and cognitive construction of academic norms (Aijaz et al., 2025). From an institutional perspective, academic ethics course experience, through systematic training and feedback mechanisms, help students develop relatively stable cognitive frameworks of integrity and strengthen their value endorsement of normative standards. Empirical evidence confirms that ethics education significantly promotes moral cognition (Bickley and Torgler, 2023).

Moreover, individuals with higher levels of moral cognition are more likely to engage in principled trade-offs under normative conflict or ambiguous conditions and to generate defensible justifications, thereby demonstrating higher-quality AI ethical decision-making (Reinecke et al., 2023). Accordingly, this study posits that moral cognition serves as a key mediating mechanism linking external normative cues to AI ethical decision-making.

The following hypotheses are proposed:

*H3*: AI ethics guidance has a significant positive effect on moral cognition.

*H4*: Academic ethics course experience has a significant positive effect on moral cognition.

*H5*: Moral cognition has a significant positive effect on AI ethical decision-making.

*H6*: Academic ethics course experience indirectly influences AI ethical decision-making through moral cognition.

*H7*: AI ethics guidance indirectly influences AI ethical decision-making through moral cognition.

#### 2.3.4 The moderating role of cognitive complexity

Cognitive complexity refers to individual differences in the capacity to differentiate multiple cues, integrate diverse perspectives, and engage in reflective trade-offs during information processing. In academic contexts where generative AI is involved, ethical decision-making frequently entails conflicts among efficiency, normative compliance, and responsibility, thereby imposing higher demands on information integration and principled reasoning. Prior research indicates that students with higher cognitive complexity are more likely to engage in deep processing of normative information and to produce more cautious and defensible judgments in complex situations (Zhai et al., 2025).

Within institutional settings, the case analyses and normative explanations provided in academic ethics course experience require substantial cognitive integration. Empirical findings suggest that students with higher cognitive complexity exhibit greater improvement in decision performance following ethics education (Rana et al., 2025). In technological environments, AI ethics guidance often presents multiple prompts and explanatory cues that likewise depend on individuals’ information integration capacity. Students with higher cognitive complexity are more likely to incorporate system prompts into a coherent evaluative framework, thereby amplifying the positive effects of AI ethics guidance (Ma et al., 2025).

Accordingly, this study proposes that cognitive complexity positively moderates the effects of academic ethics course experience and AI ethics guidance on AI ethical decision-making.

*H8*: Cognitive complexity positively moderates the relationship between academic ethics course experience and AI ethical decision-making.

*H9*: Cognitive complexity positively moderates the relationship between AI ethics guidance and AI ethical decision-making.

## 3 Method

### 3.1 Questionnaire design

This study employed a structured questionnaire to collect data. The instrument consisted of six sections: demographic information, AI ethics guidance, academic ethics course experience, cognitive complexity, moral cognition, and AI ethical decision-making. Except for demographic variables and AI ethical decision-making, all scale items were adapted from established measurement frameworks or classical conceptual models in prior research. Translation and back-translation procedures were conducted, and expert evaluation of content validity was performed to ensure semantic equivalence and contextual appropriateness.

AI ethics guidance was designed to assess students perceived normative reminders, verification prompts, and enforcement constraints when using generative AI. Item development was informed by institutional recommendations and frameworks on responsible AI use and AI use disclosure, as well as prior research on prompt-based and reminder-based interventions in academic integrity contexts, to ensure alignment with authentic learning scenarios (Cotton et al., 2024; Holmes and Miao, 2023; Kasneci et al., 2023). Academic ethics course experience measured the extent to which students engaged in case-based learning, received actionable guidance, and obtained formative feedback within ethics-related coursework. Items were adapted from pedagogical approaches that conceptualize academic integrity as a teaching and learning issue and emphasize educational intervention strategies (Gallant, 2008; McCabe and Trevino, 1993).

Cognitive complexity was adapted from established cognitive flexibility measurement frameworks and comprised six items, including one reverse-coded item that was recoded during data processing (Martin and Rubin, 1995). Moral cognition was intended to capture individuals’ recognition of moral issues and their sustained attention to and reflection upon ethical information. Items were adapted from measurement approaches in research on moral awareness and moral attentiveness (Reynolds, 2006, 2008). Except for the scenario-based tasks, all scale items were measured using a five-point Likert scale ranging from 1, strongly disagree, to 5, strongly agree. See Appendix 5 for questionnaire details.

To mitigate limitations associated with purely self-reported assessments of ethical judgment performance, AI ethical decision-making was operationalized through scenario-based tasks and modeled as a latent construct in the structural equation model, with five scenario tasks serving as reflective indicators. This design follows the situational assessment approach commonly adopted in moral judgment and moral decision-making research, in which dilemma-based or norm-conflict materials are used to elicit judgments and evaluate the quality of reasoning (Lind, 2000; Rest et al., 1999). The five scenario scores were modeled as reflective indicators because they were designed to represent different observable manifestations of the same underlying construct, namely students’ AI ethical decision-making in academic contexts. The scenarios cover different task situations, but they are expected to reflect a common latent capability rather than form separate components of the construct.

Specifically, AI ethical decision-making comprised five academic integrity-related scenarios covering AI paraphrasing and attribution, AI-generated references, AI-generated code or assignment authorship, data handling, and group work involving improper AI use. For each task, respondents were required to select the most appropriate behavioral option and provide a brief written justification. To improve transparency and replicability, the complete scenarios, scoring rubric, scoring keys, and sample responses are provided in Appendix 5.

A two-part scoring procedure was applied to each scenario. The first component assessed behavioral choice quality, scored from 0 to 2, where 2 indicated the most appropriate option, 1 indicated a partially appropriate but suboptimal choice, and 0 indicated an inappropriate choice. The second component evaluated justification quality, scored from 0 to 3, where 3 reflected principled, actionable reasoning addressing key normative considerations, 2 indicated recognition of the primary ethical issue with incomplete or insufficiently operational reasoning, 1 represented general or superficial reasoning, and 0 indicated no justification or irrelevant reasoning.

The two components were summed to obtain a raw task score ranging from 0 to 5. The raw scores were retained for analysis because they directly reflected the scoring rubric and avoided imposing an additional linear transformation on the scenario-based performance scores. In the PLS-SEM analysis, the five scenario scores were modeled as reflective indicators of AI ethical decision-making. Because standardized loadings and path coefficients were estimated and reported in the model, it was not necessary to rescale the scenario scores to match the 1–5 response format of the Likert-type constructs. Before formal scoring, two raters were trained using the scoring rubric and sample responses. The two raters then independently scored the written justifications. Inter-rater reliability was assessed using the intraclass correlation coefficient for task scores. Disagreements were resolved through discussion according to the predefined rubric. The inter-rater reliability results are reported in the Results section.

### 3.2 Data collection and sample characteristics

Data were collected through a questionnaire survey. The target population consisted of undergraduate students enrolled in Chinese universities, primarily aged 18 years and above. The inclusion criteria were as follows: participants had to be undergraduate students enrolled at participating universities, aged 18 years or above, able to understand the questionnaire content, and willing to provide informed consent before participation. The sample covered students from different academic years, disciplinary backgrounds, and levels of AI usage. To balance representativeness and feasibility, a combination of stratified sampling and convenience sampling was employed. Universities were first stratified according to geographic region, institutional type, and educational tier to reflect the major structural characteristics of China’s higher education system. Within each stratum, convenience sampling was conducted through collaboration with faculty members, classroom recruitment, and dissemination through online student communities to enhance accessibility and participation across diverse institutional and disciplinary contexts. The study involved universities from ten provinces, including Guangxi, Guizhou, and Shandong, and covered more than thirty higher education institutions to ensure regional and institutional diversity.

The questionnaire was administered through both online and offline channels. Online distribution was conducted via the Wenjuanxing survey platform, where links and QR codes were disseminated through course groups and student organization networks at participating universities. Offline data collection was conducted in public compulsory courses and major-specific classes at collaborating institutions. The same informed consent information was provided before participation in both online and offline modes. Data collection lasted approximately 3 months, from March 21 to June 26, 2025. The survey was scheduled primarily during the mid-semester period to minimize potential influences associated with semester adjustment phases or final examination preparation.

A total of 1,470 questionnaires were collected. Before analysis, all responses were screened for eligibility, completeness, and response quality. Missing data were handled through listwise deletion at the questionnaire level. Responses were excluded if they contained missing values in any focal construct item, demographic variable used in the analysis, or scenario-based AI ethical decision-making task. No statistical imputation was performed because the retained dataset consisted only of complete responses for all variables included in the analyses.

Response quality was further assessed using completion time and response-pattern checks. Questionnaires were removed if they showed abnormal completion time, defined as completion in less than one-third of the sample median completion time recorded by the survey platform, or if they displayed straight-lining patterns, defined as selecting the same response option for 20 or more of the 24 Likert-type construct items, corresponding to more than 80% of these items. In addition, responses with incomplete, blank, or invalid written justifications in the scenario-based tasks were excluded because these tasks served as performance-based indicators of AI ethical decision-making.

Based on these screening rules, 364 invalid responses were removed from the 1,470 collected questionnaires, leaving 1,106 complete and valid questionnaires for analysis. This sample size satisfies the requirements of partial least squares structural equation modeling in terms of parameter estimation stability and statistical power, and also provides sufficient case coverage for subsequent configurational analysis (Ragin, 2009; Sarstedt et al., 2021).

Table 1 presents the demographic characteristics of the sample. Gender distribution was relatively balanced, with 49.3 percent male and 50.7 percent female participants. The sample included students from different academic years and disciplinary categories, with first-year students and natural science majors constituting the largest groups. Overall, most respondents reported experience with generative AI and relatively frequent daily use, providing a suitable empirical basis for examining AI ethical decision-making.

**Table 1:** Demographic characteristics.

Category	Group	Frequency	Percentage (%)
Gender	Male	545	49.3
	Female	561	50.7
Grade	Freshman	362	32.7
	Sophomore	325	29.4
	Junior	227	20.5
	Senior	192	17.4
Major	Humanities	338	30.6
	Science	515	46.6
	Engineering	253	22.8
AI usage frequency	0 times/day	204	18.4
	1 times/day	449	40.6
	2–4 times/day	264	23.9
	≥5 times/day	189	17.1

### 3.3 Ethical statement and informed consent

This study employed an anonymous, low-risk questionnaire survey. The participants were adult undergraduate students from Chinese universities. According to Article 32 of the Measures for Ethical Review of Life Science and Medical Research Involving Humans issued by the National Health Commission of the People’s Republic of China in 2023, research using anonymous data that poses no harm and does not involve sensitive personal information or commercial interests may be exempt from ethical review. Based on this regulation, the present study was considered eligible for exemption because it collected anonymous questionnaire data only, involved minimal risk, did not collect directly identifiable information, and did not involve sensitive personal information or commercial interests. The study followed relevant ethical principles and guidelines for research involving human participants, including the Declaration of Helsinki.

All participants received the same informed consent information before completing the questionnaire. They were informed of the study purpose, the voluntary nature of participation, the right to withdraw, data usage, and privacy protection measures. For online responses, participants indicated consent electronically before entering the questionnaire. For offline responses, participants provide verbal consent before completing the paper questionnaire. Data collection was conducted from March 21 to June 26, 2025. No personally identifiable information was collected. All questionnaires were completed anonymously and analyzed in aggregate form, and all participants were adults.

### 3.4 Analytical strategy

This study adopts a complementary analytical framework combining partial least squares structural equation modeling, fuzzy-set qualitative comparative analysis, and importance–performance matrix analysis. These three methods were used sequentially and served different analytical purposes. PLS-SEM was used to estimate the net effects among latent constructs, including direct, mediating, and moderating effects. fsQCA was used to examine whether high AI ethical decision-making can be achieved through multiple combinations of conditions, thereby complementing the symmetric net-effect logic of PLS-SEM with a configurational perspective. Finally, IPMA was conducted based on the PLS-SEM results to identify antecedents and indicators with high importance but relatively low performance, thereby translating statistical results into practical intervention priorities. Together, these methods provide complementary evidence on linear associations, configurational pathways, and action priorities (Sarstedt et al., 2021).

Gender, grade, major, and frequency of AI use were included as control variables because students’ AI ethical decision-making may vary according to demographic background, academic stage, disciplinary context, and prior AI exposure. Gender was included to account for potential differences in ethical sensitivity and technology-related behavior. Grade reflected students’ academic experience and opportunities to receive formal ethics instruction. Major captured disciplinary differences in AI use, assignment types, and academic integrity norms. Frequency of AI use was included because students with greater AI exposure may have more opportunities to encounter AI-related academic integrity dilemmas. These variables were treated as control variables rather than hypothesized theoretical predictors. In the PLS-SEM model, they were entered as covariates predicting AI ethical decision-making, and the reported direct, indirect, and moderating effects were estimated after accounting for these control paths.

For measurement model assessment, this study followed established criteria for reflective constructs, comprehensively evaluating reliability, convergent validity, discriminant validity, and collinearity. The heterotrait–monotrait ratio was employed as a key criterion for discriminant validity to reduce the risk of construct overlap (Henseler et al., 2015). For structural model assessment, path coefficients, bootstrapped significance levels, coefficients of determination, effect sizes, and predictive relevance were examined. Mediation effects were tested using bootstrapping procedures to estimate indirect effects, thereby avoiding reliance on normality assumptions and enhancing inferential robustness (Preacher and Hayes, 2008). To address the potential risk of common method bias inherent in questionnaire-based research, full collinearity diagnostics were conducted as part of the robustness assessment (Kock, 2015).

Given that the formation of AI ethical decision-making exhibits characteristics of causal complexity and equifinality, a single net-effect model may be insufficient to uncover the diverse pathways through which different combinations of conditions lead to high levels of AI ethical decision-making. Therefore, this study further applied fuzzy-set qualitative comparative analysis to identify multiple sufficient configurational paths leading to high AI ethical decision-making. The explanatory power of each configuration was evaluated using consistency, raw coverage, unique coverage, and proportional reduction in inconsistency. Direct calibration was used for continuous condition variables, and the calibration anchors and thresholds are reported in the Supplementart materials. To assess the stability of the configurational results, sensitivity analyses were conducted by adjusting the frequency and consistency thresholds. This procedure complements the limitations of structural equation modeling in capturing complex causal structures (Fiss, 2011; Ragin, 2009; Schneider and Wagemann, 2012).

In addition, to enhance the practical relevance of the findings, importance–performance matrix analysis was conducted based on the partial least squares structural equation modeling results. This approach integrates the total effects of key antecedent variables with their current performance levels, allowing the identification of improvement areas with the highest intervention priority. It thereby provides actionable evidence to inform curriculum design and the optimization of AI ethics guidance (Hair and Alamer, 2022). PLS-SEM and IPMA were conducted using SmartPLS 4.0, and fsQCA was performed using the QCA package, version 3.23, in R. Descriptive statistics and correlation analyses were conducted using IBM SPSS 27.0.

## 4 Results

### 4.1 Descriptive statistics and correlation analysis

Table 2 presents the descriptive statistics of the core variables. The results indicate that the mean values of the five principal constructs are relatively high, suggesting that respondents generally reported positive perceptions or self-assessments regarding AI ethics guidance, academic ethics course experience, moral cognition, cognitive complexity, and AI ethical decision-making. Among these variables, AI ethical decision-making exhibits the highest mean value, followed by AI ethics guidance and cognitive complexity, while academic ethics course experience shows a comparatively lower mean. The standard deviations range from 0.829 to 0.900, indicating a relatively concentrated distribution with acceptable variability. These characteristics provide a sound empirical foundation for subsequent inferential analyses.

**Table 2:** Descriptive statistics and correlation analysis.

Variables	M	SD	1	2	3	4	5
1 AI ethics guidance	3.930	0.861	1				
2 Academic ethics course experience	3.882	0.900	0.577**	1			
3 Moral cognition	3.892	0.867	0.639**	0.557**	1		
4 Cognitive complexity	3.941	0.857	0.197**	0.235**	0.202**	1	
5 AI ethical decision-making	3.981	0.829	0.697**	0.692**	0.657**	0.298**	1

**p < 0.001.

Table 2 also reports the Pearson correlation matrix among the core variables. The analysis shows that all pairwise correlations are statistically significant and positive at the 0.01 level. AI ethical decision-making demonstrates the strongest correlations with AI ethics guidance and academic ethics course experience, indicating substantial associations. Moral cognition also shows relatively strong correlations with AI ethics guidance and AI ethical decision-making. In contrast, cognitive complexity exhibits comparatively lower correlation coefficients with the other variables, suggesting that although it is significantly related, the strength of association is more moderate.

### 4.2 Measurement model

After incorporating cognitive complexity as a moderating variable to construct the moderated mediation model, Appendix 2 reports the comprehensive assessment of the measurement properties. Convergent validity and reliability evaluation indicate that the standardized factor loadings of all observed items range from 0.705 to 0.792, exceeding the recommended threshold of 0.70 and confirming satisfactory construct representation. The average variance extracted values range from 0.572 to 0.609, composite reliability values range from 0.874 to 0.903, and Cronbach’s alpha coefficients range from 0.820 to 0.875. All indices surpass their respective acceptable thresholds, demonstrating strong internal consistency reliability and convergent validity. In addition, the maximum variance inflation factor across all constructs is 1.998, well below the critical value of 5.0, indicating the absence of serious multicollinearity concerns.

Regarding discriminant validity, the heterotrait–monotrait ratio results presented in Appendix 3 how that the HTMT values among all latent constructs range from 0.227 to 0.823. Cognitive complexity exhibits HTMT values below 0.30 in relation to other constructs, indicating strong discriminant validity. Although the HTMT values between AI ethical decision-making and AI ethics guidance as well as academic ethics course experience are relatively higher at 0.818 and 0.823, respectively, they remain below the recommended threshold of 0.85, thereby supporting adequate discriminant validity.

### 4.3 Structural model

Table 3 reports the overall model fit indices of the structural equation model. The R^2^ and adjusted R^2^ values for AI ethical decision-making are 0.663 and 0.660, respectively, indicating that the model explains approximately 66 percent of the variance in the dependent variable. The standardized root mean square residual is 0.039, which is below the recommended threshold of 0.08. In addition, the normed fit index is 0.901 (>0.9), suggesting a satisfactory fit between the model and the observed data. For moral cognition, the R^2^ and adjusted R^2^ values are 0.464 and 0.463, respectively. These values indicate moderate explanatory power, but they also suggest that a substantial proportion of variance in moral cognition remains unexplained. The *f* ^2^ effect sizes ranged from 0.069 to 0.285, representing decent explanatory effects of the predictors (Hair and Alamer, 2022). The Q^2^ values of all endogenous constructs fell between 0.355 and 0.435, which demonstrates the model possesses moderate predictive power (Hair and Alamer, 2022). Furthermore, the robustness of the structural model results was verified via a Bootstrapping procedure with 5,000 resamples.

**Table 3:** Model fit.

Variable	Q^2^	R^2^	R-square adjusted	SRMR	NFI
AI ethical decision-making	0.355	0.663	0.660	0.039	0.901
Moral cognition	0.367	0.464	0.463		
AI ethics guidance	0.418				
Academic ethics course experience	0.435				
Cognitive complexity	0.409				

**Figure 2:**
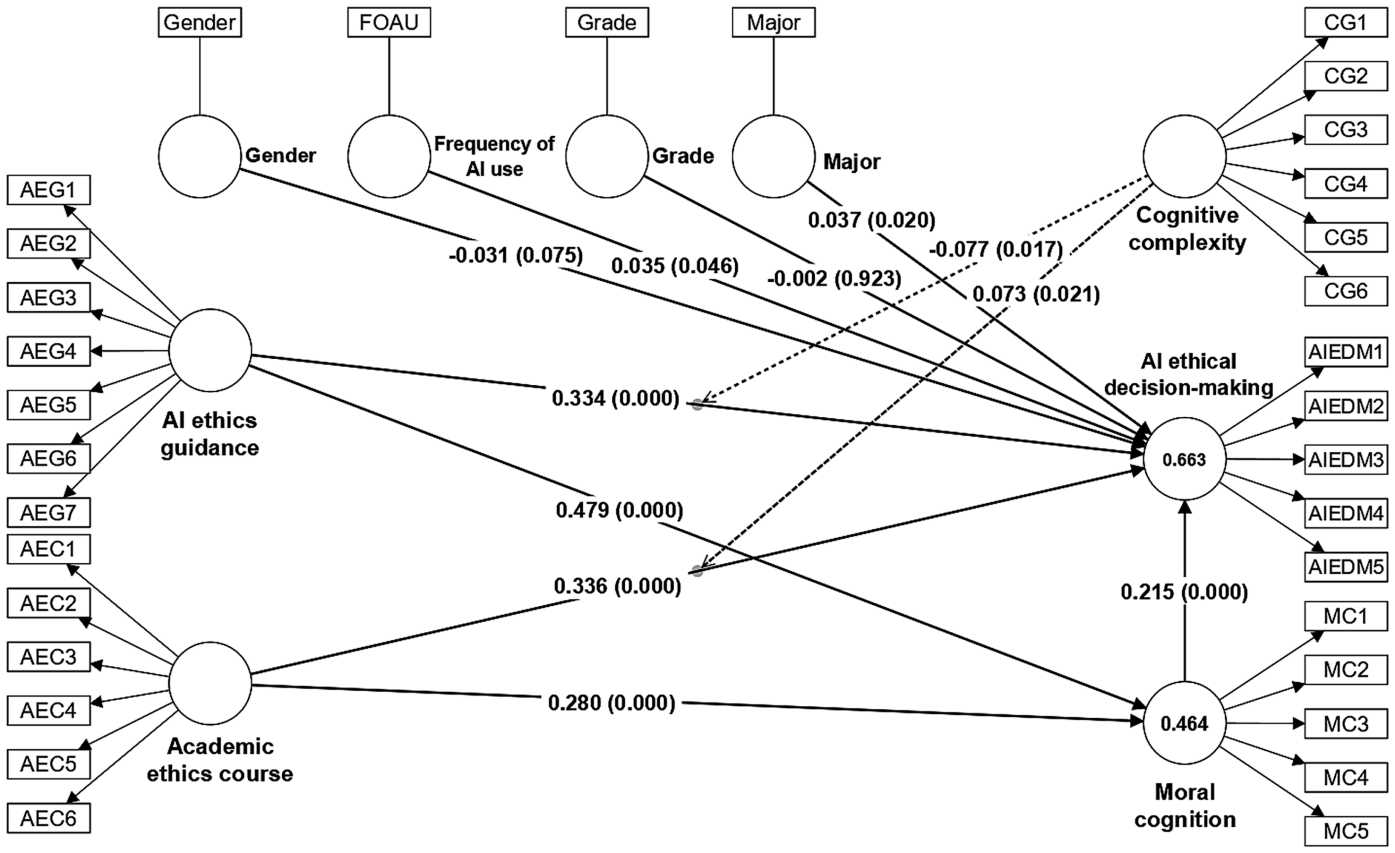
Structural equation model with mediation and moderation effects.

Figure 2 and Table 4 present the results of the path analysis. Regarding the direct effects, academic ethics course experience (*β* = 0.336, *p* < 0.001) and AI ethics guidance (*β* = 0.334, *p* < 0.001) both exert significant direct effects on AI ethical decision-making, supporting H1 and H2. In addition, academic ethics course experience (*β* = 0.280, *p* < 0.001) and AI ethics guidance (*β* = 0.479, *p* < 0.001) have significant direct effects on moral cognition. Moral cognition also demonstrates a significant direct effect on AI ethical decision-making (*β* = 0.215, *p* < 0.001). These results provide support for H3, H4, and H5.

**Table 4:** Path results of the moderated mediation model.

Hypothesis	Path	β	SD	T	P	Result
Direct effects						
H1	AEC → AIEDM	0.336	0.031	10.809	0.001	Supported
H2	AEG → AIEDM	0.334	0.035	9.466	0.001	Supported
H3	AEG → MC	0.479	0.040	12.000	0.001	Supported
H4	AEC → MC	0.280	0.039	7.205	0.001	
H5	MC → AIEDM	0.215	0.035	6.173	0.001	Supported
Indirect effects						
H6	AEC → MC → AIEDM	0.060	0.015	4.053	0.001	Supported
H7	AEG → MC → AIEDM	0.103	0.019	5.451	0.001	Supported
Moderating effects						
H8	CG x AEC → AIEDM	0.073	0.032	2.303	0.021	Supported
H9	CG x AEG → AIEDM	0.077	0.032	2.388	0.017	Supported
Control effects						
Control	Gender → AIEDM	−0.031	0.018	1.779	0.075	—
	FOAU → AIEDM	0.035	0.018	2.000	0.046	—
	Grade → AIEDM	−0.002	0.017	0.096	0.923	—
	Major → AIEDM	0.037	0.016	2.330	0.020	—

AEC: Academic ethics course experience; AEG: AI ethics guidance; AIEDM: AI ethical decision-making; MC: Moral cognition; CG: Cognitive complexity.

With respect to indirect effects, academic ethics course experience influences AI ethical decision-making through moral cognition (*β* = 0.060, *p* < 0.001), and AI ethics guidance exerts an indirect effect on AI ethical decision-making via moral cognition (*β* = 0.103, *p* < 0.001). These findings support H6 and H7.

Importantly, the moderation analysis indicates that cognitive complexity significantly and positively moderates the relationship between academic ethics course experience and AI ethical decision-making (*β* = 0.073, *p* < 0.05), supporting H8. Likewise, cognitive complexity significantly and positively moderates the relationship between AI ethics guidance and AI ethical decision-making (*β* = 0.077, *p* < 0.05), supporting H9.

The control variables were included in the same PLS-SEM model as covariates predicting AI ethical decision-making. As shown in Figure 2 and Table 4, gender was not significantly associated with AI ethical decision-making (*β* = −0.031, *p* = 0.075), and grade also showed no significant association (*β* = −0.002, *p* = 0.923). Frequency of AI use showed a small but significant positive association with AI ethical decision-making (β = 0.035, *p* = 0.046), and major also showed a small significant association (*β* = 0.037, *p* = 0.020). These control paths were included for statistical adjustment and were not treated as hypothesis tests. The direct, indirect, and moderating effects reported above were therefore estimated after controlling for gender, grade, major, and frequency of AI use.

These findings suggest that higher levels of cognitive complexity strengthen the effects of both academic ethics course experience and AI ethics guidance on AI ethical decision-making. The observed moderation effects further reveal the differentiated influence of the moderating variable on the core structural paths.

### 4.4 fsQCA

This study employed fuzzy-set qualitative comparative analysis to examine configurational pathways leading to high levels of AI ethical decision-making. Prior to the analysis, all condition variables were calibrated, except for gender, which was treated as a dichotomous variable and therefore did not require calibration (full membership: 95th percentile of the data distribution; crossover point: 50th percentile; full non-membership: 5th percentile). The calibration results indicated a satisfactory data distribution, suitable for subsequent configurational analysis. The necessity analysis results, reported in Appendix 4, show that no single condition reached the consistency threshold of 0.90; thus, no strictly necessary condition was identified. However, AI ethics guidance, academic ethics course experience, and cognitive complexity demonstrated relatively high consistency in relation to high AI ethical decision-making, suggesting that they are commonly present in most high-performing cases and therefore constitute important conditions for further configurational analysis.

Without imposing any thresholds, the initial truth table generated 49 configurations. After setting a case frequency threshold of 3, a consistency threshold of 0.80, and a proportional reduction in inconsistency threshold of 0.70, 36 configurations remained. To enhance interpretability, the proportional reduction in inconsistency threshold was further increased to above 0.85, which reduced the configurations to eight and yielded five final solutions.

Table 5 presents the multiple equifinal configurational pathways for both high and low AI ethical decision-making. The results clearly reveal asymmetry and configurational effects in the relationships between antecedent conditions and the outcome. For high AI ethical decision-making, five sufficient pathways were identified. AI ethics guidance and academic ethics course experience appear as core conditions across all high-level configurations, indicating that they constitute key structural elements in promoting high AI ethical decision-making. In contrast, moral cognition and cognitive complexity function more often as peripheral or absent conditions across different configurations, suggesting that their influence depends on specific contextual combinations rather than operating as independently decisive factors. Control variables exhibit heterogeneous patterns across configurations, indicating that high AI ethical decision-making does not depend on a single demographic characteristic but emerges from the synergistic interaction of multiple conditions.

**Table 5:** Configurational analysis.

Conditions	High AI ethical decision-making					Low AI ethical decision-making		
	1	2	3	4	5	1	2	3
AI ethics guidance	◆	◆	◆	◆	◆	●	ⓧ	●
Academic ethics course experience	◆	◆	◆	◆	◆		●	
Moral cognition	●		●		ⓧ	ⓧ		ⓧ
Cognitive complexity	●	●		ⓧ	ⓧ		ⓧ	ⓧ
Gender	ⓧ		ⓧ		●	ⓧ	ⓧ	ⓧ
Grade		ⓧ	ⓧ	●		ⓧ	ⓧ	ⓧ
Major		●		ⓧ	ⓧ	ⓧ	ⓧ	ⓧ
Frequency of AI use	●		●		ⓧ		ⓧ	
Raw coverage	0.877	0.824	0.811	0.744	0.712	0.712	0.675	0.644
Unique coverage	0.161	0.091	0.036	0.021	0.018	0.111	0.064	0.048
Consistency	0.921	0.898	0.893	0.864	0.856	0.839	0.819	0.801
Overall coverage	0.793					0.635		
Overall consistency	0.934					0.932		

◆ indicates core presence; ● indicates peripheral presence; ⓧ indicates absence of the condition; blank cells indicate that the condition may be either present or absent.

For low AI ethical decision-making, the absence of AI ethics guidance and academic ethics course experience emerges as core or important conditions, further confirming the critical inhibiting effect of ethical education elements in shaping outcomes.

Overall, the high-outcome configurations demonstrate strong explanatory power and robustness, with an overall consistency of 0.934 and overall coverage of 0.793. These findings highlight the distinct advantage of fuzzy-set qualitative comparative analysis in uncovering complex causal mechanisms and multiple equifinal pathways.

### 4.5 IPMA

**Figure 3:**
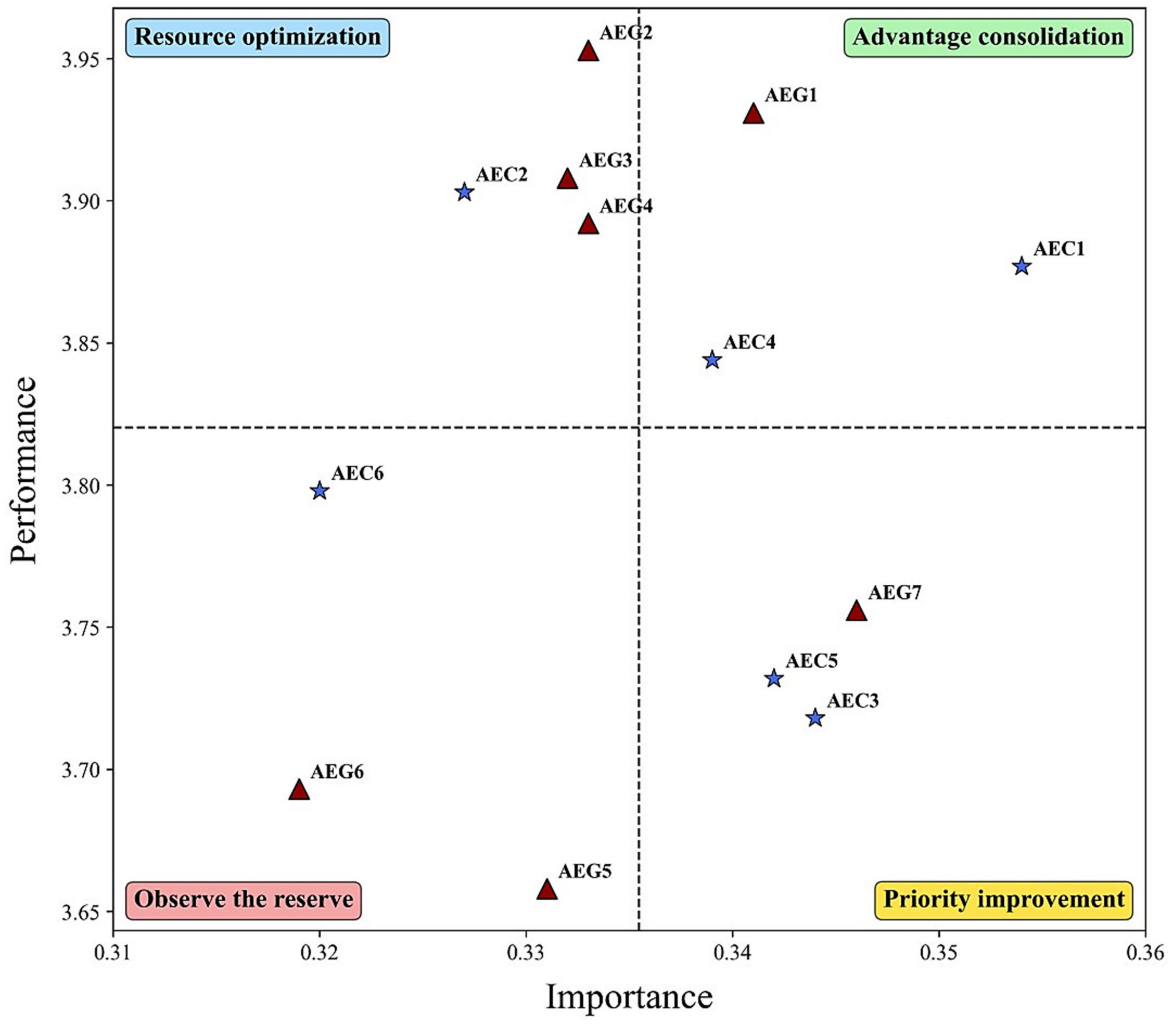
IMPA results.

To identify improvement priorities for individual items within AI ethics guidance and academic ethics course experience, this study employed importance–performance matrix analysis and constructed a four-quadrant matrix with importance on the horizontal axis and performance on the vertical axis (Figure 3). This approach simultaneously evaluates the relative impact of each element and its current performance level, thereby providing more targeted guidance for resource allocation.

The results indicate that the high-importance and high-performance quadrant includes AEG1, AEC1, and AEC4. These elements are perceived by students as important and are currently implemented effectively. They represent stable strengths within the existing AI ethics education system and should be maintained and further reinforced. The low-importance and high-performance quadrant includes AEC2, AEG2, AEG3, and AEG4. Although these elements demonstrate satisfactory performance, students assign them relatively lower priority. Resource allocation for these components may therefore be optimized to enhance efficiency.

The low-importance and low-performance quadrant includes AEC6, AEG6, and AEG5. These elements currently exert relatively limited influence and may be considered as medium- to long-term improvement areas rather than short-term priorities. In contrast, the high-importance and low-performance quadrant comprises AEC3, AEC5, and AEG7. These items are perceived as highly valuable by students but currently underperform, indicating that they constitute critical areas requiring immediate enhancement.

Overall, the importance–performance analysis suggests that improvement in AI ethics education should not rely on uniform intensification. Instead, targeted enhancement of high-importance and low-performance elements should be prioritized, while high-performance but lower-importance components may be strategically reallocated to achieve coordinated optimization of educational outcomes and resource investment.

## 5 Discussion

Situated in the context of generative AI-integrated learning, this study examined how technological and institutional normative inputs shape university students’ AI ethical decision-making. The findings show that AI ethics guidance and academic ethics course experience are both positively associated with AI ethical decision-making and also operate indirectly through moral cognition. This supports the central logic of the SOR–Social Cognitive framework: external normative inputs are not merely compliance signals but can be internalized into evaluative standards that guide students’ choices and justifications in concrete academic scenarios (Bandura, 1991; Russell and Mehrabian, 1974).

The two normative inputs operate through complementary mechanisms. AI ethics guidance provides immediate cues during AI-assisted learning and writing, such as verification reminders, citation guidance, and disclosure prompts, which are consistent with recent discussions on responsible AI use and academic integrity in higher education (Cotton et al., 2024; Kasneci et al., 2023). Academic ethics course experience provide more systematic training through case analysis, rule clarification, procedural guidance, and feedback, echoing prior work that emphasizes academic integrity as an educational and institutional responsibility rather than only a misconduct-control issue (McCabe and Trevino, 1993; Rogerson et al., 2022). Together, these findings suggest that effective AI academic integrity governance should combine real-time technological prompts with structured curricular training rather than relying on either approach alone.

The results also show that cognitive complexity strengthens the effects of AI ethics guidance and academic ethics course experience on AI ethical decision-making. This indicates that students do not respond uniformly to normative inputs. Students with stronger information integration capacity are more likely to transform external norms into decision resources when facing ambiguous AI-related academic tasks. This finding is consistent with research on integrative complexity and judgment quality, which suggests that complex decision contexts require the ability to differentiate, integrate, and weigh multiple cues (Suedfeld and Tetlock, 2014). Thus, normative interventions should consider differences in students’ cognitive processing capacity.

Finally, the fsQCA results indicate that high AI ethical decision-making can be achieved through multiple condition combinations. No single condition is strictly necessary, but AI ethics guidance and academic ethics course experience consistently occupy core positions in high-level configurations. This finding complements the PLS-SEM results by showing that AI ethical decision-making is not produced by a single linear pathway but by multiple combinations of technological, institutional, and individual conditions, which is consistent with the configurational logic of equifinality and causal asymmetry (Fiss, 2011; Ragin, 2009; Schneider and Wagemann, 2012).

There are some differences between the results of PLS-SEM and fsQCA. The PLS-SEM results indicate that cognitive complexity strengthens the average net effects of AI ethics guidance and academic ethics course experience on AI ethical decision-making. In contrast, the fsQCA results suggest that cognitive complexity is not a necessary or consistently core condition for achieving high AI ethical decision-making. This means that cognitive complexity can facilitate the translation of normative inputs into decision quality, but high decision quality may also be achieved through configurations in which strong AI ethics guidance and academic ethics course experience compensate for lower or non-core cognitive complexity. Therefore, cognitive complexity functions as an enhancing condition in the net-effect model, while remaining a substitutable or context-dependent condition in configurational pathways.

Although the proposed SOR–Social Cognitive framework specifies a theoretically ordered mechanism from normative inputs to moral cognition and AI ethical decision-making, the cross-sectional design does not allow strict causal inference. These associations are consistent with the proposed theoretical pathway, but the cross-sectional design cannot rule out reverse or reciprocal relationships. For example, students with stronger AI ethical decision-making may be more attentive to AI ethics guidance or more likely to perceive academic ethics course experience as useful. Similarly, students with higher moral cognition may report stronger sensitivity to normative inputs rather than being shaped by them alone. Moreover, the bootstrapping procedure in PLS-SEM strengthens the statistical inference of path coefficients and indirect effects by estimating their sampling stability, but it does not establish temporal ordering or eliminate unobserved confounding in cross-sectional data. Therefore, significant bootstrapped direct or indirect effects should be interpreted as theoretically guided associations rather than definitive causal evidence. Future research should test the proposed mechanism chain using longitudinal, experimental, or quasi-experimental designs. Longitudinal studies could measure AI ethics guidance, academic ethics course experience exposure, moral cognition, and AI ethical decision-making at multiple time points to examine temporal precedence and change. Experimental or quasi-experimental designs could manipulate AI ethics guidance or course-based ethics training and assess subsequent changes in moral cognition and scenario-based AI ethical decision-making.

## 6 Implications

### 6.1 Theoretical implications

The theoretical contributions of this study can be summarized in three aspects.

First, this study advances research on academic integrity in the context of generative AI from a primary focus on attitudes and intentions to AI ethical decision-making as a more context-sensitive outcome reflecting judgment quality. Prior research has largely examined whether students accept AI, perceive risks, or express compliance intentions. However, such variables are insufficient to capture variations in judgment quality within gray-area situations. The present findings indicate that the key theoretical issue in AI-era academic integrity is not merely normative endorsement, but how students identify boundaries, weigh responsibilities, and form defensible decisions in specific tasks. In doing so, this study shifts the explanatory focus from compliance tendencies to decision quality and provides a theoretical basis for incorporating situational and performance-based measures in future research.

Second, by identifying the mediating role of moral cognition, this study clarifies the psychological mechanism through which dual-source normative inputs influence AI ethical decision-making. The effect of normative input is not primarily manifested as external constraint, but rather as the transformation of norms into stable judgment competence through internalization and self-regulation. This finding extends the dominant institutional–behavioral or tool–behavioral perspectives in generative AI governance research by integrating technological prompts and curricular training within a unified internalization framework. Theoretically, AI academic integrity governance should not be conceptualized merely as rule promulgation or behavioral correction, but as a learning process that facilitates the formation of internal evaluative standards. This conclusion aligns with Social Cognitive Theory and moral psychology, which emphasize that high-quality ethical decision-making depends not only on knowing rules, but on the capacity to convert them into transferable self-regulatory standards.

Third, through the moderating effect of cognitive complexity and the integration of the Stimulus–Organism–Response and Social Cognitive frameworks, this study reveals the heterogeneity and multi-level logic underlying normative influence. The significant moderation effect suggests that normative inputs do not exert uniform effects across students; their impact depends on individuals’ information integration and evaluative capacity. This finding implies that future theoretical development should move beyond average effect explanations toward conditional perspectives that account for differences in cognitive processing resources. Furthermore, the study clarifies the functional division within the integrated framework: the Stimulus–Organism–Response structure organizes the relationships among external normative inputs, internal states, and decision outcomes, while Social Cognitive Theory explains the processes of internalization and self-regulation. This theoretical integration enhances conceptual clarity at both structural and mechanism levels.

By clarifying the distinction between moral cognition and AI ethical decision-making, this study strengthens the theoretical coherence of the proposed model. Moral cognition explains how students internalize academic integrity norms and AI-use-related ethical principles, whereas AI ethical decision-making reflects how these internalized standards are translated into defensible choices and justifications in concrete academic scenarios. This distinction separates the internalization mechanism from the decision-performance outcome and thereby improves the conceptual clarity of the SOR–Social Cognitive framework.

### 6.2 Practical implications

The practical implications focus on two priorities derived from the mechanism chain, boundary condition, and configurational findings.

First, governance efforts should promote coordination between technological and curricular approaches, upgrading from rule reminders to an integrated decision-support and training system oriented toward AI ethical decision-making. The results show that AI ethics guidance and academic ethics course experience not only directly influence AI ethical decision-making but also exert indirect effects through moral cognition. High-quality interventions should therefore serve both immediate decision support and long-term normative internalization. On the technological side, AI ethics guidance should move beyond general reminders and provide actionable, stepwise prompts at critical decision points. The importance–performance analysis indicates that citation verification prompts represent a high-priority leverage point. Emphasis should be placed on source verification, citation traceability checks, disclosure requirements, and basic robustness reporting to construct transferable self-checking procedures.

On the curricular side, academic ethics course experience should shift from rule transmission to situational and transfer-oriented training. The analysis suggests that authentic case training and concrete procedural guidance constitute priority improvement areas. Case-based learning enables students to practice responsibility trade-offs and reason generation in gray-area scenarios, while procedural guidance reduces compliance costs and facilitates the transformation of normative understanding into stable decision competence. Overall, technological prompts and curricular training are not substitutes but complementary mechanisms operating at immediate and long-term levels.

Second, intervention strategies should be stratified to account for individual differences and multi-path characteristics. The moderating role of cognitive complexity indicates that uniform intervention intensity does not yield equivalent effects across students. Normative input effectiveness depends on individuals’ cognitive integration and evaluative capacity. In practice, differentiated support should be provided according to students’ processing characteristics. For students with lower cognitive complexity, structured and stepwise support such as checklist-based verification procedures, comparative examples, and immediate feedback may reduce decision burden and enhance consistency in normative application. For students with higher cognitive complexity, open-ended case discussions, reflective writing, and situational debates may better leverage their integrative strengths and promote advanced ethical judgment.

Moreover, the configurational analysis reveals multiple pathways to high AI ethical decision-making. Accordingly, intervention design should avoid reliance on a single standardized solution and instead allow for compensatory and substitutive relationships among different elements to enhance contextual adaptability and effectiveness.

## 7 Conclusion

In the context of AI-integrated learning, this study developed and tested a psychological mechanism framework centered on AI ethical decision-making. The main conclusions are as follows: (1) AI ethics guidance and academic ethics course experience are both positively associated with university students’ AI ethical decision-making. (2) Moral cognition functions as a key internalization mechanism through which external normative inputs are linked to AI ethical decision-making. (3) Cognitive complexity strengthens the effects of AI ethics guidance and academic ethics course experience on AI ethical decision-making. (4) High AI ethical decision-making can be achieved through multiple configurations. Although no single condition is necessary, AI ethics guidance and academic ethics course experience consistently appear as core conditions in high-level pathways. The findings suggest that AI academic integrity governance should combine actionable AI-based guidance with structured academic ethics training. This study therefore advances research on academic integrity from attitudes and intentions toward scenario-based decision quality and offers practical directions for curriculum optimization and AI guidance design.

Despite these contributions, several limitations should be acknowledged. First, as discussed in the Discussion section, the cross-sectional design limits strict causal inference, and the proposed mechanism chain should be further examined through longitudinal, experimental, or quasi-experimental designs. Second, although scenario-based performance measures were used to operationalize AI ethical decision-making, the scope of scenarios may constrain generalizability. Subsequent studies could extend the scenarios to diverse disciplinary tasks and AI usage contexts. Third, institutional policy environments and curricular practices within sampled universities may affect external validity. Future research should conduct replication across institutions and cultural contexts and incorporate additional variables such as peer norms, academic pressure, moral emotions, and self-regulation strategies to refine the psychological process of normative internalization and enhance explanatory power.

## Data Availability

The original contributions presented in the study are included in the article/Supplementary material, further inquiries can be directed to the corresponding author.
