# Supplementary material for: Understanding university students’ AI ethical decision-making in academic contexts: a SOR - social cognitive perspective
Source: Front Psychol. 2026 Jul 23;17:1817904. doi: 10.3389/fpsyg.2026.1817904 (PMC13442383; doi:10.3389/fpsyg.2026.1817904)
Supplement: Supplementary file 1 [file Supplementary_File_1.docx]

Supplementary Materials

Understanding University Students’ AI Ethical Decision-Making in Academic Contexts: A SOR–Social Cognitive Perspective

**Hao Deng ^1, 2^, Minli Yang * ^3^, Liling Huang ^2^, Zhixin Yang ^2^, Nan Jiang ^2^**

^1^ Guangxi Normal University, Faculty of Education, Guangxi Guilin 541006

^2^ Guangxi Minzu University, School of Management, Guangxi Nanning 530006

^3^ Guangxi Arts University, Fine Arts College, Guangxi Nanning 530007

*** Correspondence:**

Minli Yang

[yangminli8819@126.com](mailto:yangminli8819@126.com)

**Appendix 1**

**1 Statistical Methods**

To examine both net effects and configurational effects, this study combined partial least squares structural equation modeling (PLS-SEM) and fuzzy-set qualitative comparative analysis (fsQCA). The two methods are complementary in logic and purpose. PLS-SEM is a variance-based approach that estimates linear and symmetric relationships among latent constructs and is particularly suitable for prediction-oriented models with complex paths, including mediation and moderation (Hair et al., 2019). It is also relatively robust when data deviate from multivariate normality.

To complement the symmetric analysis, fsQCA was applied to capture causal complexity and equifinality. Unlike regression-based approaches that focus on the isolated contribution of individual predictors, fsQCA adopts a configurational perspective and identifies multiple combinations of conditions associated with the same outcome (Chuah et al., 2021; Gil-Cordero et al., 2024). It is especially useful for examining nonlinearity, asymmetry, and interdependence among conditions, and for distinguishing necessary from sufficient conditions.

PLS-SEM analyses and IPMA were conducted in SmartPLS 4.0, and fsQCA analyses were performed using the QCA package (version 3.23) in R (Abbasi et al., 2022). Descriptive statistics and correlation analyses were conducted usingIn addition, to enhance the practical relevance of the findings, importance–performance matrix analysis was conducted based on the partial least squares structural equation modeling results. This approach integrates the total effects of key antecedent variables with their current performance levels, allowing the identification of improvement areas with the highest intervention priority. It thereby provides actionable evidence to inform curriculum design and the optimization of AI ethics guidance (Hair & Alamer, 2022). Descriptive statistics and correlation analyses were conducted using IBM SPSS Statistics 27.0.

**1.1 PLS-SEM procedure**

The PLS-SEM analysis followed the standard two-step procedure: assessment of the measurement model and evaluation of the structural model (Hair et al., 2019). All focal constructs in this study were modeled as reflective constructs, as the indicators were treated as manifestations of their underlying latent variables.

**Measurement model assessment**

- The measurement model was evaluated in terms of reliability, convergent validity, discriminant validity, and collinearity.
- Internal consistency reliability was assessed using Cronbach’s alpha and composite reliability (CR), with values above 0.70 indicating acceptable reliability (Hair et al., 2019).
- Convergent validity was examined using average variance extracted (AVE), with a recommended threshold of 0.50 or above (Hair et al., 2019).
- Discriminant validity was assessed using the Fornell–Larcker criterion, under which the square root of each construct’s AVE should exceed its correlations with other constructs (Abbasi et al., 2022).
- Multicollinearity was checked using variance inflation factor (VIF), with values below 5 indicating no critical collinearity concern.

**Structural model assessment**

After confirming the adequacy of the measurement model, the structural model was tested. Path significance was estimated using a bias-corrected bootstrapping procedure with 5,000 resamples and 95% confidence intervals (Hair et al., 2019). The structural model was evaluated based on:

- Path coefficients and t-values (for testing hypothesized relationships),
- Coefficient of determination (R²) (for explanatory power),
- Effect size (f²) (for the contribution of each predictor),
- Predictive relevance (Q²) (for out-of-sample predictive capability).

Following common guidelines, R² values of 0.75, 0.50, and 0.25 may be interpreted as substantial, moderate, and weak explanatory power, respectively (Hair et al., 2019). The f² statistic was used to assess the relative impact of each predictor on endogenous constructs (Welkowitz et al., 2011).

**1.2 fsQCA procedure**

FsQCA was used to further examine whether high levels of the outcome (i.e., AI ethical decision-making) can emerge from multiple combinations of causal conditions. This analysis complements PLS-SEM by focusing on configurational causation rather than net linear effects.

The fsQCA analysis involved three steps: calibration, necessity analysis, and sufficiency analysis.

**Step 1: Calibration**

Calibration transforms raw variable scores into fuzzy-set membership scores ranging from 0 to 1, where 0 indicates full non-membership, 1 indicates full membership, and intermediate values represent partial membership (Thygeson et al., 2011). In this study, direct calibration was used by specifying three qualitative anchors for each condition: full membership, crossover point, and full non-membership (Diwanji, 2023).

**Step 2: Necessity analysis**

The second step examined whether any single condition qualified as necessary for the outcome. A condition is considered necessary when the outcome is consistently a subset of that condition. Following prior studies, a consistency threshold of 0.90 was used as the benchmark for necessity (Abbasi et al., 2022; Janse van Rensburg et al., 2021).

**Step 3: Sufficiency analysis**

The final step assessed sufficient configurations by constructing a truth table and identifying combinations of conditions linked to high (and, where relevant, low) levels of the outcome. For 𝑘 causal conditions, the truth table contains 2^𝑘^ logically possible rows, each representing a distinct configuration (Huarng & Roig-Tierno, 2016). To identify robust sufficient configurations, this study applied commonly used thresholds for consistency, coverage, and proportional reduction in inconsistency (PRI), following established fsQCA guidelines (Huarng & Roig-Tierno, 2016; Janse van Rensburg et al., 2021).

In fsQCA, consistency indicates the degree to which cases conform to the set-theoretic relationship implied by a configuration, coverage reflects the empirical relevance of a configuration for explaining the outcome, and PRI helps ensure that a configuration is not simultaneously associated with both the presence and absence of the outcome.

**1.3 IPMA procedure**

In addition to PLS-SEM and fsQCA, this study employed importance–performance map analysis (IPMA) to derive more actionable implications from the structural model results. IPMA extends the standard PLS-SEM results by jointly considering (a) the importance of each construct (or indicator) for a target outcome and (b) its performance level, thereby helping identify priority areas for improvement (Hair et al., 2019).

In this study, AI ethical decision-making was specified as the target construct. The importance of each antecedent was derived from its total effect on the target construct in the PLS-SEM model, while performance was calculated from the latent variable scores (or indicator scores) rescaled to a 0–100 metric, following the IPMA procedure in SmartPLS (Ramayah et al., 2014). Constructs (and, where relevant, indicators) with high importance but relatively low performance were interpreted as priority intervention targets, because improving these elements is expected to yield the greatest gains in the target outcome.

To enhance practical interpretability, the IPMA was further examined at the indicator level for key constructs (e.g., AI ethics guidance and academic ethics course), allowing the study to pinpoint specific instructional or design elements that should be prioritized. This indicator-level diagnosis complements the construct-level PLS-SEM and the configurational insights from fsQCA by translating statistical findings into concrete optimization priorities for educational practice and AI ethics guidance design.

**Appendix 2**

Variable reliability and convergent validity

| **Variables** | **Items** | **Factor loadings** | **VIF** | **Cronbach's alpha** | **CR** | **AVE** |
| --- | --- | --- | --- | --- | --- | --- |
| Academic ethics course | AEC1 | 0.772 | 1.775 | 0.871 | 0.903 | 0.609 |
|  | AEC2 | 0.783 | 1.849 |  |  |  |
|  | AEC3 | 0.764 | 1.745 |  |  |  |
|  | AEC4 | 0.772 | 1.757 |  |  |  |
|  | AEC5 | 0.785 | 1.862 |  |  |  |
|  | AEC6 | 0.806 | 1.998 |  |  |  |
| Cognitive complexity | CG1 | 0.766 | 1.718 | 0.860 | 0.895 | 0.587 |
|  | CG2 | 0.783 | 1.741 |  |  |  |
|  | CG3 | 0.721 | 1.658 |  |  |  |
|  | CG4 | 0.767 | 1.655 |  |  |  |
|  | CG5 | 0.772 | 1.821 |  |  |  |
|  | CG6 | 0.787 | 1.93 |  |  |  |
| AI ethics guidance | AEG1 | 0.78 | 1.875 | 0.875 | 0.903 | 0.572 |
|  | AEG2 | 0.761 | 1.779 |  |  |  |
|  | AEG3 | 0.759 | 1.787 |  |  |  |
|  | AEG4 | 0.761 | 1.789 |  |  |  |
|  | AEG5 | 0.773 | 1.831 |  |  |  |
|  | AEG6 | 0.753 | 1.739 |  |  |  |
|  | AEG7 | 0.705 | 1.585 |  |  |  |
| Moral cognition | MC1 | 0.775 | 1.672 | 0.821 | 0.875 | 0.583 |
|  | MC2 | 0.766 | 1.623 |  |  |  |
|  | MC3 | 0.765 | 1.628 |  |  |  |
|  | MC4 | 0.792 | 1.692 |  |  |  |
|  | MC5 | 0.719 | 1.476 |  |  |  |
| AI ethical decision-making | AIEDM1 | 0.772 | 1.657 | 0.820 | 0.874 | 0.581 |
|  | AIEDM2 | 0.745 | 1.519 |  |  |  |
|  | AIEDM3 | 0.759 | 1.612 |  |  |  |
|  | AIEDM4 | 0.756 | 1.589 |  |  |  |
|  | AIEDM5 | 0.779 | 1.675 |  |  |  |

**Appendix 3**

Heterotrait-monotrait ratio (HTMT)

|  | **AI ethics guidance** | **Academic ethics course** | **Cognitive complexity** | **Moral cognition** | **AI ethical decision-making** |
| --- | --- | --- | --- | --- | --- |
| **AI ethics guidance** |  |  |  |  |  |
| **Academic ethics course** | 0.661 |  |  |  |  |
| **Cognitive complexity** | 0.227 | 0.271 |  |  |  |
| **Moral cognition** | 0.754 | 0.658 | 0.240 |  |  |
| **AI ethical decision-making** | 0.823 | 0.818 | 0.354 | 0.801 |  |

**Appendix 4**

Necessity analysis

|  | AI ethical decision-making | | ~ AI ethical decision-making | |
| --- | --- | --- | --- | --- |
|  | Consistency | Coverage | Consistency | Coverage |
| Gender | 0.511 | 0.552 | 0.503 | 0.448 |
| ~Gender | 0.489 | 0.544 | 0.497 | 0.456 |
| Grade | 0.590 | 0.727 | 0.612 | 0.622 |
| ~Grade | 0.693 | 0.684 | 0.731 | 0.595 |
| Major | 0.783 | 0.718 | 0.755 | 0.571 |
| ~Major | 0.532 | 0.725 | 0.628 | 0.705 |
| Frequency of AI use | 0.757 | 0.727 | 0.702 | 0.557 |
| ~Frequency of AI use | 0.539 | 0.687 | 0.656 | 0.690 |
| AI ethics guidance | 0.817 | 0.785 | 0.729 | 0.578 |
| ~AI ethics guidance | 0.561 | 0.715 | 0.729 | 0.767 |
| Academic ethics course | 0.804 | 0.809 | 0.682 | 0.566 |
| ~Academic ethics course | 0.568 | 0.685 | 0.770 | 0.765 |
| Moral cognition | 0.773 | 0.776 | 0.705 | 0.584 |
| ~Moral cognition | 0.586 | 0.707 | 0.729 | 0.726 |
| Cognitive complexity | 0.788 | 0.769 | 0.736 | 0.593 |
| ~Cognitive complexity | 0.583 | 0.728 | 0.713 | 0.735 |

**Appendix 5**

Questionnaire

**Individual characteristics**

Gender: Male; Female.

Grade: Freshman; Sophomore; Junior; Senior.

Major: Science; Humanities; Engineering.

AI usage frequency (weekly): 0 times; 1 times; 2-4 times; ≥5 times.

**AI ethics guidance**

**Dimension 1**: Reminders

1. When I use AI for coursework, the AI often reminds me about academic integrity (e.g., plagiarism, authorship, proper use).

2. The AI clearly signals when my request or intended use may violate academic norms.

**Dimension 2**: Verification

3. The AI encourages me to verify information using reliable sources rather than accepting outputs at face value.

4. The AI prompts me to provide or check citations/references when I use AI-generated content in academic work.

5. The AI reminds me to distinguish between my own ideas and material derived from external sources (including AI outputs and the sources behind them).

**Dimension 3**: Enforcement

6. The AI’s integrity guidance is strong enough that I often have to adjust my behavior (e.g., rephrase, add citations, verify sources).

7. The AI sometimes restricts or warns against risky actions (e.g., discourages direct copying or flags potential misconduct).

**Academic ethics course**

**Dimension 1**: Case-based learning

1. The course uses realistic cases (including AI-related cases) to help us judge what is acceptable vs. unacceptable academic behavior.

2. In the course, we are asked to justify our decisions in ethical/academic integrity scenarios (not just memorize rules).

**Dimension 2**: Actionable guidance

3. The course provides clear, practical guidelines for handling citations, attribution, and disclosure (including AI-assisted work).

4. The course teaches concrete steps I can apply (e.g., how to document sources, verify claims, and disclose AI use when required).

**Dimension 3**: Assessment/Feedback

5. The course assesses academic integrity in a meaningful way (e.g., scenario-based tasks, reflections, or applied exercises).

6. The course provides feedback that helps me correct integrity-related mistakes (e.g., citation practice, source verification, AI use boundaries).

**Cognitive complexity**

1. When facing complex issues, I tend to analyze from multiple perspectives rather than focusing on a single factor.

2. I can understand conflicting viewpoints and synthesize them into a more comprehensive judgment.

3. Before making decisions, I consider both short-term and long-term consequences of different options.

4. I am willing to revise my conclusions when new information emerges.

5. I can integrate rules, context, and personal goals rather than applying rules mechanically.

6. I prefer simple, clear answers and am reluctant to deal with complex or ambiguous issues. (Reverse scoring)

**Moral cognition**

1. I can clearly distinguish appropriate from inappropriate AI use in academic tasks.

2. When making academic decisions, I proactively consider integrity principles rather than convenience alone.

3. I endorse academic integrity norms and view them as internal standards I should follow.

4. Even without external monitoring, I tend to choose the more integrity-consistent option.

5. I can explain why certain misconduct is wrong.

**AI Ethical Decision-Making Scenarios**

AI ethical decision-making was assessed using five scenario-based tasks. Each task required respondents to select the most appropriate behavioral option and provide a brief written justification. The behavioral option captured decision choice quality, while the written justification captured reasoning quality in scenario responses. Together, the five scenario scores were used as reflective indicators of the latent construct of AI ethical decision-making.

**Task 1**: AI paraphrasing and attribution

**Scenario**: You use an AI tool to fully paraphrase a passage relevant to your assignment into a polished paragraph that you could insert directly. You did not copy exact sentences, but the core ideas come from several published articles.

**Question**: What is the most appropriate action?

A. Use the paragraph without citation because it is not verbatim copying.

B. Use the paragraph, cite the original sources, and disclose AI assistance if required by course policy.

C. Do not use the paragraph; rewrite everything entirely on your own.

**Justification** (2–5 sentences): __________________________

**Task 2**: AI-generated references of uncertain authenticity

**Scenario**: The AI generates several references that look credible, but you are short on time and have not verified whether they actually exist.

**Question**: What is the most appropriate action?

A. Include the references anyway; they are probably real.

B. Keep only references you can verify as real; remove or replace any unverifiable ones.

C. Keep them but add a note saying “AI-generated references, not verified.”

**Justification** (2–5 sentences): __________________________

**Task 3**: AI-generated code/assignment and academic authorship

**Scenario**: A course requires you to submit a piece of code (or a written report). You use AI to generate most of it. You understand the general logic, but you cannot fully explain every line/section.

**Question**: What is the most appropriate action?

A. Submit it as your own work because you “basically understand it.”

B. Validate and revise the output, ensure you can explain it, and disclose AI assistance if permitted; if AI use is not permitted, use AI only for learning and do not submit AI-generated content.

C. AI use should always be prohibited, regardless of course policy.

**Justification** (2–5 sentences): __________________________

**Task 4**: Data handling and the temptation of “better results”

**Scenario**: You analyze your data and your key result is not statistically significant. You notice that removing a few “outliers” makes the result significant, but those outliers are not clearly erroneous observations.

**Question**: What is the most appropriate action?

A. Remove the outliers to obtain significance; the paper will be more acceptable.

B. Apply a justified, transparent rule for handling outliers and report robustness results with and without those observations.

C. Report only the significant results and omit details about data handling.

**Justification** (2–5 sentences): __________________________

**Task 5**: Group work and unequal/improper AI use

**Scenario**: In a group project, one member heavily uses AI to generate content and directly pastes it into the final submission. You worry this could expose the whole group to academic integrity risk, but the member says, “Everyone does it.”

**Question**: What is the most appropriate action?

A. Ignore it—getting a good grade comes first.

B. Communicate with the group and align the work with course rules (e.g., verification, citation, disclosure, revision); if uncertainty remains, consult the instructor about acceptable boundaries.

C. Immediately report the member and refuse to participate.

**Justification** (2–5 sentences): __________________________

**Scoring Rubric for AI Ethical Decision-Making Scenarios**

Each scenario was scored using two components: behavioral choice quality and justification quality. The behavioral choice was scored from 0 to 2, and the written justification was scored from 0 to 3. The two components were summed to obtain a raw score ranging from 0 to 5 for each scenario. The raw score was then transformed to a 1-to-5 scale using the following formula: 1 + 4 × (raw score / 5).

**Behavioral choice quality**:

0 = inappropriate choice that violates or ignores academic integrity requirements.

1 = partially appropriate but incomplete or suboptimal choice.

2 = most appropriate choice that aligns with academic integrity requirements.

**Justification quality**:

0 = no justification, irrelevant justification, or justification that supports misconduct.

1 = general or superficial justification without clear reference to academic integrity principles.

2 = recognition of the main ethical issue, but with incomplete or insufficiently actionable reasoning.

3 = principled and actionable reasoning that addresses key normative considerations, such as attribution, verification, disclosure, transparency, responsibility, or consultation with the instructor when boundaries are unclear.

**Scoring Keys for Behavioral Choices**

**Task 1**: AI paraphrasing and attribution

A = 0; B = 2; C = 1.

**Task 2**: AI-generated references of uncertain authenticity

A = 0; B = 2; C = 1.

**Task 3**: AI-generated code/assignment and academic authorship

A = 0; B = 2; C = 1.

**Task 4**: Data handling and the temptation of “better results”

A = 0; B = 2; C = 0.

**Task 5**: Group work and unequal/improper AI use

A = 0; B = 2; C = 1.

**Sample Responses for Justification Scoring**

The following examples illustrate how written justifications were scored. These examples are based on the attribution scenario and were used to guide rater training.

**Score 3 example**:

“I would verify the sources, cite the original materials, and disclose AI assistance if the course policy requires it. Even if the AI-generated text is not copied word for word, the core ideas come from external sources, so attribution and transparency are necessary.”

**Score 2 example**:

“I should cite the sources because the ideas are not completely mine, but I may still use the AI-polished paragraph after some revision.”

**Score 1 example**:

“It is better to be careful when using AI because academic rules are important.”

**Score 0 example**:

“I would use the AI paragraph directly because it sounds original and the wording is different.”

**References**

Abbasi, G. A., Sandran, T., Ganesan, Y., & Iranmanesh, M. (2022). Go cashless! Determinants of continuance intention to use E-wallet apps: A hybrid approach using PLS-SEM and fsQCA. *Technology in Society*, *68*, 101937.

Chuah, S. H.-W., Tseng, M.-L., Wu, K.-J., & Cheng, C.-F. (2021). Factors influencing the adoption of sharing economy in B2B context in China: Findings from PLS-SEM and fsQCA. *Resources, Conservation and Recycling*, *175*, 105892.

Diwanji, V. S. (2023). Fuzzy‐set qualitative comparative analysis in consumer research: A systematic literature review. *International Journal of Consumer Studies*, *47*(6), 2767-2789.

Gil-Cordero, E., Ledesma-Chaves, P., Arteaga Sánchez, R., & Mariano, A. M. (2024). Crypto-wallets revolution! Key factors driving behavioral intention to adopt the Coinbase Wallet using mixed PLS-SEM/fsQCA methodology in the Spanish environment. *International Journal of Bank Marketing*, *42*(3), 536-570.

Hair, J. F., Risher, J. J., Sarstedt, M., & Ringle, C. M. (2019). When to use and how to report the results of PLS-SEM. *European Business Review*, *31*(1), 2-24.

Huarng, K.-H., & Roig-Tierno, N. (2016). Qualitative comparative analysis, crisp and fuzzy sets in knowledge and innovation. *Journal of Business Research*, *69*(11), 5181-5186.

Janse van Rensburg, A., Kathree, T., Breuer, E., Selohilwe, O., Mntambo, N., Petrus, R.,…Petersen, I. (2021). Fuzzy-set qualitative comparative analysis of implementation outcomes in an integrated mental healthcare trial in South Africa. *Global health action*, *14*(1), 1940761.

Ramayah, T., Chiun, L. M., Rouibah, K., & May, O. S. (2014). Identifying Priority Using an Importance-Performance Matrix Analysis (IPMA): The Case of Internet Banking in Malaysia. *International Journal of E-Adoption*, *6*(1), 1-15.

Thygeson, N. M., Solberg, L. I., Asche, S. E., Fontaine, P., Pawlson, L. G., & Scholle, S. H. (2011). Using fuzzy set qualitative comparative analysis (fs/QCA) to explore the relationship between medical “homeness” and quality. *Health services research*, *47*(1 Pt 1), 22.

Welkowitz, J., Cohen, B. H., & Lea, R. B. (2011). *Introductory statistics for the behavioral sciences*. John Wiley & Sons.
